# Prevalence and correlates of objectively measured weight status among urban and rural Mozambican primary schoolchildren: A cross-sectional study

**DOI:** 10.1371/journal.pone.0228592

**Published:** 2020-02-03

**Authors:** Taru Manyanga, Joel D. Barnes, Jean-Philippe Chaput, Lise Dubois, Peter T. Katzmarzyk, Emily F. Mire, Antonio Prista, Mark S. Tremblay

**Affiliations:** 1 Healthy Active Living and Obesity Research Group, CHEO Research Institute, Ottawa, Canada; 2 School of Epidemiology and Public Health, Faculty of Medicine, University of Ottawa, Ottawa, Canada; 3 Pennington Biomedical Research Center, Baton Rouge, Louisiana, United States of America; 4 Research Group for Physical Activity and Health (CIDAF-FEFD), Universidade Pedagógica, Maputo, Mozambique; University of Maiduguri College of Medical Sciences, NIGERIA

## Abstract

**Background:**

The coexistence of undernutrition (thinness) and overnutrition (overweight/obesity) among children and adolescents is a public health concern in low-middle-income countries. Accurate prevalence estimates of thinness and overweight/obesity among children and adolescents are unavailable in many low-middle-income countries due to lack of data. Here we describe the prevalences and examine correlates of objectively measured weight status among urban and rural schoolchildren in Mozambique.

**Methods:**

A cross-sectional study design was applied to recruit 9-11-year-old schoolchildren (n = 683) from 17 urban and rural primary schools in Mozambique. Body mass index (BMI) was computed from objectively measured height and weight and participants’ weight categories were determined using the World Health Organization cut-points. Actigraph GT3X + accelerometers were worn 24 hours per day for 7 days to assess movement behaviours. Multilevel multivariable modelling was conducted to estimate odds ratios and confidence intervals.

**Results:**

Combined prevalence of overweight/obesity (11.4%) was significantly higher among urban participants compared to rural participants (5.7%; χ^2^ = 7.1; p = 0.008). Conversely, thinness was more prevalent among rural (6.3%) compared to urban (4.2%) participants. Passive school commute, not meeting daily moderate- to vigorous-intensity physical activity (MVPA) guidelines, and maternal BMI >25 kg/m^2^ were associated with overweight/obesity while possessing one or more functional cars at home, maternal BMI >25 kg/m^2^ and being an older participant were associated with thinness in the present sample. The proportion of total variance in the prevalences of obesity and/or thinness occurring at the school level was 8.7% and 8.3%, respectively.

**Conclusion:**

Prevalences of thinness, overweight/obesity and other key variables differ between urban and rural schoolchildren in Mozambique. MVPA, active transport and mother’s BMI are important modifiable correlates of weight status among Mozambican schoolchildren. Results from this study demonstrate important differences between urban and rural schoolchildren that should not be ignored when designing interventions to manage malnutrition, formulating public health strategies, and interpreting findings.

## Background

Prevalence estimates from the World Health Organization (WHO) show that childhood obesity is increasing globally [1]. Although some studies in high-income countries (HICs) have reported a recent plateau [2,3], obesity levels remain high among all children and youth [3]. Moreover, in low-middle-income countries (LMICs), childhood obesity is reportedly increasing [4], and at a much faster pace than it did in HICs [5,6]. Obesity is linked to numerous non-communicable diseases (NCDs) [1], is known to track from childhood to adulthood [7,8] and is associated with a higher risk of premature mortality [9]. In LMICs, undernutrition (underweight/thinness, wasting, stunting) also still persists. Undernutrition is linked to health consequences such as suboptimal adult health (e.g. delayed pubertal maturation, premature mortality, increased susceptibility to fat accumulation mostly in the central region of the body, insulin resistance) [10,11], poor cognitive and motor development, and has negative consequences for work productivity, thus ultimately perpetuating poverty [12,13]. The coexistence of undernutrition and overnutrition (overweight, obesity), commonly referred to as the double burden of malnutrition [6,14], is challenging for LMICs that still face high prevalences of infectious diseases [15]. Recently, the WHO declared childhood obesity as one of the greatest challenges of the 21st century, announcing that ending it was a top priority [1]. However, many LMICs face the double burden of communicable diseases and NCDs [16], and have limited resources to enable them to adequately address these competing public health priorities.

Despite the well-known differences in lifestyle behaviours between urban and rural populations [17,18], there is limited available evidence in LMICs [19,20] where research is still mostly based on urban and non-representative samples [21]. Relying on this evidence alone to inform public health policies or to design interventions may be ill-informed and inappropriate. Furthermore, behavioural and environmental factors (e.g. home or school environments) that may be associated with the weight status of schoolchildren in LMICs such as Mozambique are not well documented. The most recent synthesis of data on key indicators of physical activity among Mozambican children and youth identified addressing the absence of such evidence as an urgent priority [22]. Not surprisingly, this lack of data is most apparent in the rural areas of Mozambique.

In the present study, multilevel multivariable models were used to examine the prevalence and correlates of objectively measured weight status among urban and rural schoolchildren in Mozambique. We hypothesized that the prevalence of unhealthy weight status (thinness, overweight/obese) would significantly differ between urban and rural schoolchildren. We further hypothesized that the correlates of thinness or overweight/obesity would also be different between urban and rural schoolchildren.

## Methods

### Study design

This is a cross-sectional study of a sex-balanced (i.e., proportionate number of boys and girls) non-nationally representative sample (n = 683) of 9–11 year-old primary schoolchildren recruited from 10 urban (Maputo, stratified by socioeconomic status) and 7 rural (Macia district) schools in Mozambique. At least three urban schools were recruited from each of three districts, using a list provided by the Ministry of Education to maximize variability in levels of neighborhood socioeconomic status (SES). Rural schools were conveniently recruited from a list provided by the district education office. One urban school declined to participate after being approached and was replaced by another school from the same district. In this study, we followed the published protocol and methodology used in the International Study of Childhood Obesity, Lifestyle and the Environment (ISCOLE) [23]. Recruited schools were used as the primary sampling framework. Once a school had consented to participate, students in grades four and five (likely to be closest to age 10) were targeted for recruitment, were given an introduction letter and consent forms for their parents/guardians to review and either consent or decline. The consent forms provided details on the study protocol as well the data to be collected, including the sociodemographic data needed from the parents as part of the study. Data were collected throughout the Mozambican primary school year between August 2017 and May 2018.

### Ethics approvals

Prior to data collection, ethical approvals were received from the Children’s Hospital of Eastern Ontario Research Ethics Board (#17/59X), the University of Ottawa Research Ethics Board (#A05-17-02), and the Mozambique National Bioethics Committee for Health (#151/CNBS/17). Written informed consent was obtained from parents or legal guardians before including participants in this study. Parental (Demographic and Health Questionnaire; Neighbourhood Environment) questionnaires were only given to parents after they had consented for their children to participate. For parents/guardians with low levels of literacy, research assistants who were fluent in Portuguese (the official language), and the native language (Xangana), read and explained the contents of consent forms. Parents/guardians for rural participants were invited to the school for consent and parental questionnaires where a trained interviewer individually helped them to complete the questionnaires. In developing this study, we adhered to the Strengthening the Reporting of Observational Studies in Epidemiology (STROBE) guidelines for cross-sectional studies [24].

## Measures

### Anthropometric measurements

Participants’ standing height was measured to the nearest 0.1 cm by trained and certified research staff using a Seca 213 portable stadiometer (Seca Corporation, Hamburg, Germany). With the participant standing as erect as possible and with their head in the Frankfort horizontal plane, measurements were recorded at the end of a deep inhalation. The participant’s body weight was measured to the nearest 0.5 kg using a portable Tanita Body Composition Analyzer (SC-240, Illinois, USA) after all outer clothing, heavy pocket items, shoes, and socks were removed. All measurements were done in duplicate, and a third measurement was taken if the first two were more than 0.1 cm (height), or 0.5 kg (body weight) apart. The average of the closest two measurements was used for analyses. BMI (kg/m^2^) was derived from standing height and body weight, and converted to BMI z-scores using sex- and age-adjusted growth references developed by the WHO for 5–19 year-old children and youth [25,26]. The WHO defined cut-points were applied to classify participants as being thin (BMI z-scores < -2 standard deviations [SD]), healthy weight (BMI z-scores between -2 and +1 SD), overweight (BMI z-scores > +1 to +2 SD), or obese (BMI z-scores > +2 SD) [25,26]. Participants parents/guardians were classified as underweight (BMI < 18.5 kg/m^2^), healthy weight (18.5–42.9 kg.m^2^), overweight (25.0–29.9 kg.m^2^), and obese (≥30.0 kg/m^2^) [27].

### Movement behavior measurements

Actigraph GT3X+ accelerometers were used to objectively measure nocturnal sleep, total sedentary time, and physical activity. The accelerometers were attached to an elastic belt and worn lying flat on the right hip, for seven consecutive days plus an initial familiarization day. To improve compliance (i.e. minimize episodes of forgetting to wear accelerometers in the morning), a 24-hour protocol was implemented, encouraging participants to wear the accelerometer at all times except during water based activities [28]. An algorithm was used to identify total nocturnal sleep time and an additional algorithm was used to identify periods of awake non-wear time (any sequence of ≥20 consecutive minutes of 0 activity counts) [29]. Data were collected at a sampling rate of 80 Hz, downloaded in 1-second epochs with the low-frequency extension filter using the ActiLife software version 6.5.4 (ActiGraph LLC, Pensacola, FL, USA). Data were subsequently aggregated to 15- and 60-second epochs for summarizing physical activity and nocturnal sleep, respectively. After accounting for the total nocturnal sleep and awake non-wear time [29], all remaining minutes were classified as awake-wear time. Participants with ≥10 hours of wear time per day on at least 4 days including 1 weekend day were considered to have sufficient physical activity data. Cut-points developed by Evenson et al. [30] were used to quantify sedentary time (≤25 counts), light physical activity (LPA) (26–573 counts), moderate physical activity (574–1002 counts) and vigorous physical activity (≥1003 counts) per 15-second epochs.

### Questionnaires

Participants were asked to complete a *Diet and Lifestyle Questionnaire* related to their dietary intake, physical activity, sedentary behaviours and sleep patterns, in the presence of research staff during a school visit. Parents/guardians completed a *Demographic and Health Questionnaire* which captured the participant’s health history; parental education, number of siblings, parental weight and height, household income; and home food environment information. Parents/guardians also completed a *Neighbourhood Environment Questionnaire* which captured information about parental perceptions of their neighborhoods’ social, built, food and physical activity environment. A school administrator completed the *School Environment Questionnaire* capturing information about the participants’ school characteristics, policies and practices that could potentially influence participants’ healthy eating and physical activity behaviours. Items on the questionnaires were validated and were successfully used in the ISCOLE [23]. Questionnaires were forward and backward translated, and specific items on the questionnaires were adapted to reflect local contexts. Complete questionnaires are available elsewhere [23].

### Covariates

Participants’ sex and age, and the highest level of parental education (a proxy of SES), and school location (urban/rural) were used as covariates in all multivariable models because of the plausibility of confounding.

### Sample size

Sample size calculations were partially guided by those done for ISCOLE [23]. Assuming: 1) that participants would be recruited in clusters with an average of 25 students per school; 2) approximately 5.3% difference in obesity prevalence between urban (6.8%) [ref. 23] and rural (1.5%) [31] primary schoolchildren; and 3) at least 80% power, a total of 444 participants for both urban and rural (222 each) would be required. To account for the cluster sampling, we estimated a design effect of 1.3, resulting in a required sample size of 578; and anticipating approximately 10% of participants to have invalid and/or incomplete data, the recruitment target was 650 students.

### Treatment of missing data

Overall, 103 participants (15%) were missing data on the highest level of parental education and 149 participants (22%) had insufficient accelerometry data. Participants with missing data did not significantly differ in mean age (mean difference = 0.02; p = 0.7), BMI z-scores (mean difference = -0.13; p = 0.10) or sex (chi-square = 1.31; p = 0.3). The proportion of participants missing data on the highest level of parental education did not differ from those with complete data (p = 0.7). To minimize loss of information, and potentially biasing the results due to excluding missing cases [32], multiple imputation by chained equations (MICE) was applied [33] using the R statistical Package, “mice”[34]. Missing values were multiply imputed (50 datasets) under the Missing at Random (MAR) assumptions [33]. Before assuming that data were MAR, model-based recursive partitioning analysis [35], the Little MCAR’s test [36], and missing patterns analyses were performed [33].

### Statistical analyses

Statistical analyses were computed using SAS 9.4 (SAS Institute Inc., North Carolina, USA) and R (version 3.5.2; The R Foundation for Statistical Computing, Vienna, Austria). Descriptive characteristics of participants were summarized using means (SD) or frequencies (percentages) as appropriate. Unpaired t-tests and chi-square tests (χ^2^) were used to examine potential differences between participants attending urban versus rural schools. Multilevel multivariable logit models (PROC GLIMMIX) accounting for clustering at the school level were used to determine the correlates of participants’ weight status (thinness versus non-thin or overweight/obese versus non-overweight/obese). For analyses, the non-thin category included participants classified as healthy weight plus overweight/obese. The non-overweight/obese category included those classified as thin and healthy weight. Schools were treated as random effects in all models. Potential correlates of thinness or overweight/obesity were selected *a priori*, based on previous literature [37–41] and the modified socioecological model proposed by Davison & Birch [42]. Correlates included directly measured and reported variables obtained from questionnaire data. Table 1 presents the list of potential correlates and how they were used in the analyses. First, each potential correlate was included in univariable models and those that were at least marginally (p < 0.10) statistically significant were retained for use in the multivariable models. This less-strict criterion was applied for univariable analyses to prevent the potential exclusion of important variables [43]. Potential correlates that remained marginally statistically significant (p<0.10) from the univariable analyses were entered in final models including all variables and covariates. Variables that were statistically significant (p<0.05) in the final models were considered to be correlates of overweight/obesity or thinness.

**Table 1:** Potential correlates of objectively measured thinness or overweight/obesity.

Variable	Method of measurement	Use in analysis
**Individual characteristics**		
Sex	Parent-reported	Binary variable: male or female (covariate)
Age	Parent-reported	Continuous (covariate)
School commute (mode of transport to and from school for main part of the journey)	Participant-reported	Re-coded as a dichotomous variable: active (walking, bicycle/rollerblade/skateboard/scooter), or passive (bus/train/ boat, car/motorcycle/moped)
Total sedentary time	Accelerometer measured	Continuous
Light-intensity physical activity	Accelerometer measured	Continuous
Moderate-to-vigorous-intensity physical activity	Accelerometer measured	Continuous: re-coded as dichotomous (< 60 minutes per day) or (≥60 minutes per day)
Sleep duration	Accelerometer measured	Continuous
Recreational screen time	Participant-reported	Continuous
Outdoor play time (before school, after school, weekend)	Participant-reported	Continuous
Recreational screen time	Participant-reported	Dichotomous: (≤ 2 hours of recreational screen time) or (> 2 hours of recreational screen time) per day
Participation in sports	Participant-reported	Dichotomous: (Did not participate in sporting activities) or (participated in sporting activities) in the past year
Health-related quality of life	Participant-reported	Dichotomous: (poor, fair) or (good, very good, excellent)
Consumption of breakfast	Participant-reported	Dichotomous: (eats breakfast ≤ 6 days per week) or (eats breakfast daily)
Consumption of fruits	Participant-reported	Dichotomous: (eats fruits ≤ 3 days per week) or (eats fruits 4 or more days per week)
Consumption of vegetables	Participant-reported	Dichotomous: (eats vegetables ≤ 3 days per week) or (eats vegetables 4 or more days per week)
Consumption of fast-food	Participant-reported	Dichotomous: (eats fast food ≤ 3 times per week) or (eats fast food > 3 times per week)
Consumption of fried food	Participant-reported	Dichotomous: (eats fried food ≤ 3 times per week) or (eats fried food > 3 times per week)
Consumption of fries	Participant-reported	Dichotomous: (eats fries ≤ 3 times per week) or (eats fries > 3 times per week)
Consumption of fast food while watching television	Participant-reported	Dichotomous (Does not eat fast food while watching television) or (eats fast food while watching television at least once per week)
**Characteristics of participant’s parents/legal guardians**		
Mother’s BMI	Parent-reported	Continuous: re-coded as dichotomous <25 or ≥25 kg/m^2^
Father’s BMI	Parent-reported	Continuous: re-coded as dichotomous <25 or ≥25 kg/m^2^
Parental level of education	Parent-reported	Re-coded as highest level of parental education (covariate): <high school, high school/some college, or bachelor’s/graduate degree
Mother’s work status	Parent-reported	Continuous: re-coded as dichotomous (≤15 hours/week) or (>15 hours/week)
Father’s work status	Parent-reported	Continuous: recoded as dichotomous (≤15 hours/week) or (>15 hours/week)
**Home Environment**		
Number of televisions in the house	Parent-reported	Re-coded as categorical: 0 or 1 or ≥2
Number of functional cars at home	Parent-reported	Re-coded as categorical: 0 or 1 or ≥2
Number of siblings for participant	Parent-reported	Continuous: recoded as dichotomous ≤2 or ≥3
Number of residents at home	Parent-reported	Continuous: recoded as dichotomous; ≤3 or≥4
**Neighborhood Environment**		
High crime rate in the neighbourhood	Parent-reported	Re-coded as binary: “disagreed/strongly disagreed”, and “agreed/strongly agreed”
Trust people in the community	Parent-reported	Re-coded as binary: “disagreed/strongly disagreed”, and “agreed/strongly agreed”
**School Environment**		
School location	School-Administrator-reported	Binary: urban or rural
Physical activity policies	School-Administrator-reported	Binary: yes/no
Healthy eating policies	School-Administrator-reported	Binary: yes/no

BMI: Body Mass Index

For intercept estimates to be more meaningful, all continuous variables were grand mean centered prior to estimating the models. Denominator degrees of freedom, were calculated using the Kenward Roger approximation (DDFM = KR) [44]. Variance tolerance inflation factors (VIF) were applied to test for multicollinearity in multivariable models [45]. Unless stated (i.e. VIFs > 5), no problems regarding multicollinearity were identified. Covariance parameter estimates from an unconditional model (i.e., a model with the dependent variable only) were used to compute Intraclass Correlation Coefficients (ICC) indicating how much of the total variance in the prevalences of overweight/obesity or thinness was attributed to individuals (level 1) or schools (level 2).

Sensitivity analyses were conducted to compare univariable results from imputed data sets to those from complete case analyses, and results were similar. Because the results from complete case analysis and multiply imputed data analyses were similar, the imputed data were used for final analyses to maximize data utilization. Unbalanced weight status (i.e. low prevalence of o overweight/obesity among rural participants) within the models made it impractical to separately estimate models for rural and urban sub-samples. However, because of important descriptive differences between the two sub-samples, density curves for key variables were plotted. In addition, the location of school (rural or urban) was added to multivariable models as an additional covariate.

## Results

Table 2 presents the descriptive characteristics of the participants with complete data, stratified by rural or urban school location. The whole sample (52.9% girls) had a mean age of 10.1 ± 0.8 years, and BMI z-score of -0.4 ± 1.0. Apart from sex (χ^2^ = 0.8; p = 0.4), all other descriptive characteristics (age, BMI z-scores, weight status, levels of parental education) were statistically significantly different between urban and rural participants. The ICC obtained from unconditional multilevel models showed that the proportion of total variance in the prevalences of overweight/obesity or thinness occurring at the school level was 8.7% and 8.3% respectively. The remaining proportion of variability in each of overweight/obesity or thinness was explained by individual or other unmeasured factors. Combined prevalence of overweight/obesity (11.4%) was significantly higher among urban participants compared to rural participants (5.7%; χ^2^ = 7.1; p = 0.008). Conversely, thinness was more prevalent among rural (6.3%) compared to urban (4.2%) participants. More boys (6.8%) than girls (3.9%) were thin. Virtually all rural schoolchildren (98.3%) reported that they actively commuted to school while 69.1% of urban schoolchildren used active transport. Rural schoolchildren had an average of 19 more minutes of LPA, and 24 more minutes of moderate- to vigorous- intensity physical activity (MVPA) per day than those attending urban schools.

**Table 2:** Descriptive characteristics (n = 683).

**Continuous variables**	**Mean (SD)**				
	**Total sample**	**Urban**	**Rural**	**t-value**	**p-value**
Age (years)	10.1 (0.8)	10.2 (0.8)	10.1 (0.8)	2.5	0.01*
BMI z-score	-0.4 (1.0)	-0.2 (1.0)	-0.5 (0.9)	3.3	0.001*
**Categorical variables**	**N (%)**			**Chi-Square**	**p-value**
Sex (% female)	683 (52.9)	333 (54.7)	350 (51.1)	0.8	0.4
**Weight Categories**					
Thinness	36 (5.3)	14 (4.2)	22 (6.3)	8.7	0.03*
Normal Weight	589 (86.2)	281 (84.4)	308 (88.0)		
Overweight	46 (6.7)	29 (8.7)	17 (4.9)		
Obese	12 (1.8)	9 (2.7)	3 (0.9)		
Parents did not complete high school	431 (74.3)	172 (62.1)	259 (85.5)	48.8	< .0001*
Parents completed high school, some college	111 (19.1)	71 (25.6)	40 (13.2)		
Parents completed bachelors or higher degree	38 (6.6)	34 (12.3)	4 (1.3)		

Data are presented for participants with complete data. SD: Standard Deviation; BMI: Body Mass Index

* = statistically significant at p < 0.05

**Fig 1:**
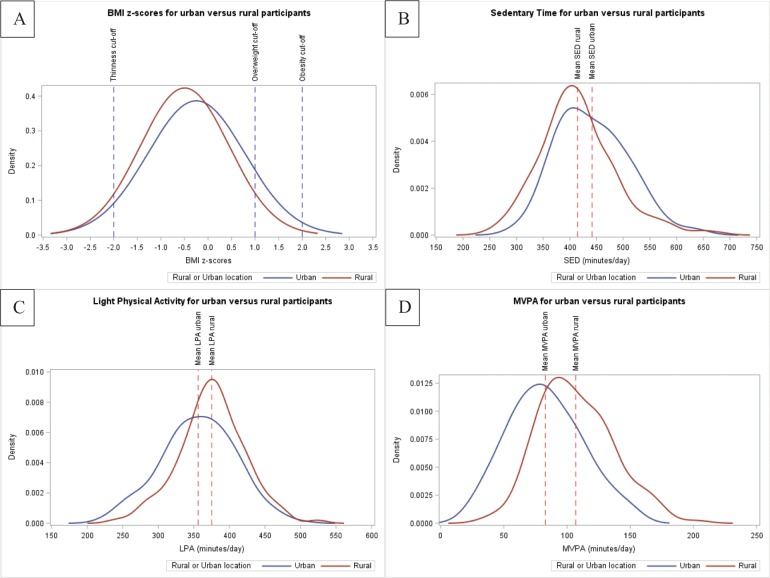
Density curves based on complete cases. Density curves based on complete cases, comparing body mass index z-scores (A), total sedentary time (B), light physical activity (C), and moderate-vigorous-physical activity (D), between urban and rural participants. BMI = Body Mass Index; SED = Total Sedentary Time; LPA = Light Physical Activity; MVPA = Moderate- to Vigorous- Intensity Physical Activity.

Density curves presented in Fig 1 compare BMI z-scores (panel A), sedentary time (SED) (panel B), LPA (panel C), and MVPA (panel D) between participants from urban and rural schools and illustrate distinct differences. Tables 3 and 4 separately present the odds ratios for the relationships between overweight/obesity, and thinness and each of their potential correlates. Of the 33 potential correlates, 10 had a marginal (p <0.10) statistically significant association with overweight/obesity and five were significantly associated with thinness in the univariable analyses. Most (passive commute to school, not meeting MVPA guidelines, higher recreational screen time, frequent consumption of fast food, frequent consumption of fast food while watching TV, mother’s BMI >25 kg/m^2^, father works ≥15 hours/week, one or more cars at home, urban school) were positively associated with overweight/obesity while having three or more siblings was the only variable inversely associated with overweight/obesity. Frequent consumption of fast food, mother’s BMI >25 kg/m^2^, and having one or more cars at home were inversely associated with thinness while being male and an older participant were positively associated with being thin.

**Table 3:** Univariate correlates of overweight/obesity.

Variables	Estimate	SE	Odds Ratio	Confidence limits	p-value
**Individual characteristics**					
Sex (ref: boys)	0.11	0.28	1.12	0.64–1.93	0.7
Age	0.32	0.20	1.38	0.94–2.03	0.1
Commute to school (ref: active)	1.21	0.34	3.37	1.72–6.60	0.0006*
Total sedentary time	0.00	0.00	1.00	1.00–1.00	0.8
Light-intensity physical activity	0.00	0.00	1.00	0.99–1.00	0.7
MVPA (ref: meeting guidelines)	1.00	0.38	2.71	1.29–5.73	0.009*
Sleep duration	0.00	0.00	1.00	0.99–1.01	0.4
Recreational screen time	0.15	0.06	1.16	1.03–1.30	0.01*
Outdoor playtime	-0.02	0.10	0.98	0.81–1.18	0.8
Participation in sports (ref: participated in sporting activities in past year)	0.43	0.34	1.54	0.79–3.00	0.2
Health-related quality of life (ref: excellent)	0.27	0.33	1.30	0.68–2.49	0.4
Consumption of breakfast (ref: ≤ 6 days/ week)	-0.01	0.36	0.99	0.49–1.99	0.9
Consumption of fruits (ref: ≤ 3 days/week)	0.34	0.29	1.42	0.79–2.52	0.2
Consumption of vegetables (ref: ≤ 3 days/week)	-0.03	0.31	0.97	0.52–1.79	0.9
Consumption of fast-food (ref: ≤3 times/ week)	0.82	0.32	2.27	1.22–4.24	0.01*
Consumption of fried food (ref: ≤ 3 days/week)	0.34	0.29	1.41	0.80–2.50	0.2
Consumption of fast food while watching television (ref: does not eat food watching TV)	0.73	0.31	2.08	1.12–3.84	0.02*
**Characteristics of participant’s parents/legal guardians**					
Mother’s BMI (ref: <25 kg/m^2^)	1.41	0.39	4.10	1.92–8.75	0.0003*
Father’s BMI (ref: <25 kg/m^2^)	0.17	0.29	1.18	0.66–2.11	0.6
Level of parental education (ref: <high school, high school/some college)	0.50	0.30	1.65	0.90–2.99	0.1
Mother works (ref: ≤15 hours/week)	0.36	0.30	1.44	0.80–2.59	0.2
Father works (ref: ≤15 hours/week)	0.72	0.31	2.05	1.11–3.77	0.02*
**Home Environment**					
Number of televisions in the house (ref:<2)	0.09	0.32	1.08	0.59–2.00	0.8
Number of functional cars at home (ref:<2)	0.50	0.29	1.64	0.93–2.93	0.09*
Number of participant’s siblings (ref: ≤2)	-0.53	0.28	0.59	0.34–1.03	0.06*
Number of residents at home (ref: ≤3)	-0.34	0.30	0.70	0.39–1.27	0.2
**Neighborhood Environment**					
High crime rate in the neighbourhood (ref: disagree)	0.33	0.31	1.38	0.76–2.52	0.3
Trust people in the community (ref: disagree)	0.19	0.29	1.21	0.69–2.13	0.5
**School Environment**					
School location (ref: rural)	-0.74	0.37	0.48	0.21–1.04	0.06*
Physical activity policies (ref: yes)	0.31	0.41	1.37	0.57–3.30	0.4
Healthy eating policies (ref: yes)	0.09	0.41	1.10	0.46–2.63	0.8

MVPA: moderate- to vigorous-intensity physical activity; BMI: body mass index

* statistically significant at p < 0.05

**Table 4:** Univariate correlates of thinness.

Variables	Estimate	SE	Odds Ratio	Confidence limits	p-value
**Individual characteristics**					
Sex (ref: boys)	-0.64	0.35	0.53	0.26–1.06	0.07*
Age	0.52	0.24	1.69	1.05–2.70	0.03*
Commute to school (ref: active)	-0.31	0.59	0.73	0.23–2.33	0.6
Total sedentary time	0.00	0.00	1.00	0.99–1.01	0.6
Light-intensity physical activity	0.00	0.00	1.00	0.99–1.00	0.4
MVPA (ref: meeting guidelines)	0.66	0.53	1.93	0.68–5.44	0.2
Sleep duration	0.00	0.00	1.00	0.99–1.01	0.5
Recreational screen time	-0.13	0.09	0.88	0.74–1.04	0.1
Outdoor playtime	-0.07	0.13	0.94	0.72–1.21	0.6
Participation in sports (ref: participated in sporting activities in past year)	0.17	0.43	1.18	0.51–2.73	0.7
Health-related quality of life (ref: excellent)	-0.21	0.47	0.81	0.32–2.05	0.7
Consumption of breakfast (ref: ≤ 6 days/ week)	-0.53	0.39	0.59	0.27–1.26	0.2
Consumption of fruits (ref: ≤ 3 days/week)	-0.30	0.39	0.73	0.34–1.60	0.4
Consumption of vegetables (ref: ≤ 3 days/week)	-0.57	0.47	0.56	0.22–1.42	0.2
Consumption of fast-food (ref: ≤3 times/ week)	-1.04	0.63	0.35	0.10–1.21	0.09*
Consumption of fried food (ref: ≤ 3 days/week)	0.06	0.38	1.06	0.50–2.25	0.9
Consumption of fast food while watching television (ref: does not eat food watching TV)	-0.29	0.42	0.75	0.32–1.72	0.5
**Characteristics of participant’s parents/legal guardians**					
Mother’s BMI (ref: <25 kg/m^2^)	-0.93	0.36	0.39	0.20–0.80	0.009*
Father’s BMI (ref: <25 kg/m^2^)	0.46	0.38	1.59	0.75–3.32	0.2
Level of parental education (ref: <high school, high school/some college)	0.24	0.41	1.27	0.57–2.85	0.5
Mother works (ref: ≤15 hours/week)	0.08	0.39	1.08	0.50–2.33	0.8
Father works (ref: ≤15 hours/week)	0.27	0.37	1.31	0.64–2.70	0.5
**Home Environment**					
Number of televisions in the house (ref:<2)	-0.39	0.39	0.68	0.31–1.48	0.3
Number of functional cars at home (ref:<2)	-0.88	0.38	0.42	0.20–0.87	0.02*
Number of participant’s siblings (ref: ≤2)	-0.11	0.36	0.90	0.44–1.83	0.8
Number of residents at home (ref: ≤3)	-0.16	0.36	0.85	0.43–1.71	0.7
**Neighborhood Environment**					
High crime rate in the neighbourhood (ref: disagree)	-0.00	0.37	0.99	0.48–2.04	0.9
Trust people in the community (ref: disagree)	0.45	0.36	1.57	0.78–3.15	0.2
**School Environment**					
School location (ref: rural)	0.37	0.46	1.45	0.56–3.77	0.4
Physical activity policies (ref: yes)	-0.38	0.49	0.68	0.25–1.86	0.4
Healthy eating policies (ref: yes)	0.15	0.46	1.16	0.44–3.05	0.8

MVPA: moderate- to vigorous-intensity physical activity; BMI: body mass index

*: statistically significant at p < 0.05

Table 5 presents results of the final multivariable models that included all marginally statistically significant variables from univariable analyses and the covariates. Three variables (passive commute to school, not meeting daily MVPA guidelines, and having a mother with BMI >25 kg/m^2^) were associated with increased odds of being overweight/obese. Having one or more functional cars at the home (inverse), mother’s BMI >25 kg/m^2^ (inverse) and being an older participant (positive) were associated with, and were identified as correlates of thinness. Mother’s BMI >25 kg/m^2^ (inversely related to thinness and positively related to overweight/obesity) was the only variable that remained significant for both thinness and overweight/obesity in final multivariable regression models. As shown in supplementary files, S1 Fig, density curves for BMI z-scores, SED, and LPA are not very different between boys and girls while boys have higher MVPA than girls. S2 and S3 Figs present density curves that compare similar variables and confirm the differences in key variables for urban and rural boys and girls separately. S1 Table presents aggregate descriptive data for all variables used in the analytic data-set.

**Table 5:** Final multivariable models.

Variables	Estimate	SE	Odds Ratio	Confidence limits	p-value
**Correlates of overweight/obesity**					
Commute to school (ref: active)	0.86	0.38	2.36	1.11–5.00	0.03*
MVPA (ref: meeting guidelines)	0.98	0.43	2.68	1.15–6.18	0.02*
Recreational screen time	0.05	0.07	1.06	0.92–1.21	0.4
Consumption of fast-food (ref: ≤3 times per week)	0.65	0.38	1.92	0.92–4.02	0.08
Consumption of fast food while watching television (ref: does not eat)	0.48	0.42	1.61	0.74–3.65	0.3
Mother’s BMI (ref: <25 kg/m^2^)	1.61	0.40	4.94	2.26–10.94	< .0001*
Father works (ref: ≤15 hours/week)	0.54	0.33	1.72	0.90–3.28	0.1
Number of participant’s siblings (ref: ≤2)	-0.50	0.30	0.61	0.34–1.10	0.1
School location (ref: rural)	0.46	0.50	1.59	0.58–4.32	0.4
Sex (ref: boys)	0.06	0.31	1.06	0.58–1.92	0.9
Age	0.40	0.21	1.50	0.99–2.28	0.06
Level of parental education (ref: <high school, high school/some college)	-0.00	0.31	0.99	0.51–1.92	0.9
**Correlates of thinness**					
Mother’s BMI (ref: <25 kg/m^2^)	-1.13	0.37	0.33	0.16–0.68	0.003*
Functional cars at home (ref: ≤1)	-1.18	0.42	0.30	0.14–0.70	0.005*
Consumption of fast-food (ref: ≤3 times per week)	-1.00	0.67	0.37	0.10–1.36	0.1
School location (ref: rural)	0.72	0.53	2.02	0.68–6.01	0.2
Sex (ref: boys)	-0.70	0.37	0.50	0.24–1.02	0.06
Age	0.56	0.25	1.75	1.06–2.87	0.03*
Level of parental education (ref: <high school, high school/some college)	0.20	0.45	1.23	0.51–2.94	0.6

MVPA: moderate- to vigorous-intensity physical activity; BMI: body mass index; SES: Socioeconomic status

*: statistically significant at p < 0.05

Models were adjusted for age, sex, SES, school location

## Discussion

This study examined the prevalence and correlates of thinness or overweight/obesity among urban and rural schoolchildren in Mozambique. The results indicate that overweight/obesity is higher among urban, while thinness is higher among rural, schoolchildren. With the exception of one variable (mother’s BMI), our findings show different correlates for thinness and overweight/obesity. Passive commuting to school, not meeting daily MVPA guidelines and having a mother with overweight/obesity are statistically significant correlates of overweight /obesity. Having one or more cars at home, mother’s BMI >25 kg/m^2^, and older age are statistically significant correlates of thinness. Results from this study illustrate distinct differences in key variables between urban and rural Mozambican primary schoolchildren. The emergence of obesity and the persistence of thinness as serious public health concerns among children and adolescents in LMICs necessitate prioritizing effective and evidence-informed prevention and management strategies.

The presence of both thinness and overweight/obesity in this study validates the need to consider examining both forms of malnutrition when designing studies in samples such as the present one. The present findings confirm results from previous studies [6,12,31,46,47] that document the existence of the dual burden of malnutrition among children and adolescents in LMICs. While prudent for public health strategies to appropriately continue focusing on undernutrition, present and previous findings [46] show that overweight/obesity, especially among urban Mozambican schoolchildren is increasing, and needs attention. Finding a high prevalence of overweight/obesity among urban Mozambican schoolchildren and thinness among rural schoolchildren supports our primary hypothesis. These findings are comparable to those reported by Gomes et al. [18], albeit in an adult sample and demonstrate important differences between urban and rural populations in Mozambique. These key differences must be considered, and should compel researchers to include rural participants in similar studies conducted in LMICs such as Mozambique, that still have large proportions of the population living in the rural areas. Consistent with our findings, Nhantumbo et al [48] previously reported a higher prevalence of undernutrition and negligible overweight status in a sample of rural Mozambican children and adolescents. These results suggest that public health strategies should be dually focused and aim to address thinness and overweight/obesity separately for rural and urban schoolchildren.

MVPA [40] and active transport [49,50] are important lifestyle correlates of overweight/obesity among children. In previous multinational studies involving LMICs and HICs, objectively measured low MVPA has been consistently shown to be associated with obesity [40,51,52]. Consistent with results from a previous Mozambican [53] and other international studies [51,52], our findings presented in S1 Fig show that on average, boys (red curve) accumulate more minutes of daily MVPA than girls (blue curve). This finding demonstrates that this difference between boys and girls is not unique to one region or country and may be related to cultural expectations or contextual factors that require further exploration to enable interventions facilitating equitable participation for girls. These present findings further support the robustness of MVPA as an important correlate of obesity in many different contexts. Previous results for active transportation are equivocal [49,54], suggesting that its association with obesity may be context-specific or may be related to the limitations of self-reported data.

Findings from the present study identifying both active transport and high MVPA as protective correlates for overweight/obesity among schoolchildren suggest that promoting these lifestyle behaviours as effective and less expensive strategies for active healthy living, especially in resource-limited LMICs may yield positive public health outcomes. However, it is also plausible (because the cross-sectional design cannot establish temporality), that it is indeed the overweight/obesity status that may be driving the lower MVPA or passive transport commute among participants with overweight/obesity. Higher proportions of active school commuters and more MVPA minutes among rural schoolchildren than those from urban schools suggest ongoing physical activity transitions [55] among urban-dwellers in Mozambique and demonstrates the overall importance of utilitarian physical activity. Although low prevalence of overweight/obesity among rural participants made it impractical to estimate separate models for rural and urban schoolchildren, descriptive results presented in density curves support our secondary hypothesis and further corroborates the need to include rural and urban participants in future studies.

High maternal BMI was the only significant correlate, though in opposite directions, for both overweight/obesity and thinness in the present study. This finding showing that higher maternal BMI was positively and negatively associated with overweight/obesity and thinness, respectively, is consistent with findings from previous studies [56,57] and may be indicative of the important role that mothers play in shaping lifestyle choices for their children, especially in the context of LMICs. It is also plausible to speculate that this finding may be related to household food insecurity where mothers would overconsume poor-quality diet leading to overweight, the child consumes small amount of the same food leading to undernutrition. Having one or more functional cars at home might have been a proxy for family affluence, hence its significant inverse relationship with thinness in the present study. Unlike overweight/obesity, thinness among children in LMICs may be driven by higher-order risk factors, e.g. poverty or food security which were not measured in the present study [58–60]. Future studies among populations in similar settings may need to consider measuring the potential influences of such variables. Previous studies have found socioeconomic status [61,62] and older participant’s age [62,63] to be correlates of thinness among children of this age group. Because thinness, which measures the nutritional status of children above five years of age and adolescents, is generally not an indicator prioritized globally [62], it is rarely monitored nor reported, hence the few available comparable data.

This study has several limitations, including its cross-sectional design which precludes inferences about causation. Relationships found, are limited to the list of included correlates and we also cannot exclude the potential confounding effects of unmeasured variables. The study sample is non-representative, and for several variables, relied on self-reported data obtained by questionnaires whose validity in this context, especially for the rural population, is not known. There were substantial data missing on key variables which necessitated the application of multiple imputations with potential biases that may have been introduced. However, comparative analyses between complete data cases and imputed datasets do not support this potential limitation. We used BMI z-score cut-points developed from a different reference population which may have resulted in inadvertently over or underestimating prevalences of thinness or overweight/obesity. Finally, we did not measure food insecurity which is known associate with thinness in children. Nonetheless, this study has important strengths that include the recruitment of a relatively large sample involving both urban and rural participants. We objectively measured anthropometry and movement behaviours, and followed a rigorous and standardized study protocol. For example, research staff were trained and certified prior to data collection. Finally, our multilevel analyses accounted for the hierarchical nature of these data.

## Conclusion

Prevalences of thinness and overweight/obesity and other key variables (SED, LPA, MVPA, active transport) differed between urban and rural schoolchildren in Mozambique. Active transport and MVPA were statistically significant correlates of overweight/obesity while having one or more functional cars at home and being older were significant correlates for thinness. High maternal BMI was a significant correlate for both thinness (negative) and overweight/obesity (positive) albeit in opposing directions. Results from this study demonstrate important differences between urban and rural schoolchildren that should not be ignored when designing interventions to prevent all forms of malnutrition (over and undernutrition), formulating public health strategies, and interpreting findings.

## Supporting information

S1 Table Aggregate descriptive data for variables in analytic dataset. (DOCX)

S1 Fig Density curves based on complete cases, comparing body mass index z-scores (A), total sedentary time (B), light physical activity (C), and moderate-vigorous-physical activity (D), between boys and girls. BMI = Body Mass Index; SED = Total Sedentary Time; LPA = Light Physical Activity; MVPA = Moderate- to Vigorous- Intensity Physical Activity.
(EPS)

S2 Fig Density curves based on complete cases, comparing body mass index z-scores (A), total sedentary time (B), light physical activity (C), and moderate-vigorous-physical activity (D), between urban and rural boys. BMI = Body Mass Index; SED = Total Sedentary Time; LPA = Light Physical Activity; MVPA = Moderate- to Vigorous- Intensity Physical Activity.
(EPS)

S3 Fig Density curves based on complete cases, comparing body mass index z-scores (A), total sedentary time (B), light physical activity (C), and moderate-vigorous-physical activity (D), between urban and rural girls. BMI = Body Mass Index; SED = Total Sedentary Time; LPA = Light Physical Activity; MVPA = Moderate- to Vigorous- Intensity Physical Activity.
(EPS)
