# Supplementary material for: Prevalence and correlates of objectively measured weight status among urban and rural Mozambican primary schoolchildren: A cross-sectional study
Source: PLoS One. 2020 Feb 3;15(2):e0228592. doi: 10.1371/journal.pone.0228592 (PMC6996844; doi:10.1371/journal.pone.0228592)
Supplement: S1 Table — (DOCX) [file pone.0228592.s001.docx]

**S1 Table: Aggregate descriptive data for variables in analytic dataset (n=683)**

| **Variable** | **Mean** | **SD** | **Range** | **Kurtosis** | **Skewness** |
| --- | --- | --- | --- | --- | --- |
| Sex | 1.5 | 0.5 | 1.0 | -0.2 | -0.1 |
| Age (years) | 10.1 | 0.8 | 3.5 | -0.8 | -0.2 |
| BMI z-score | -0.4 | 1.0 | 6.9 | 0.4 | 0.2 |
| SED (minutes) | 428.6 | 63.7 | 445.9 | 1.1 | 0.5 |
| LAP (minutes) | 364.6 | 44.9 | 305.2 | 0.8 | 0.01 |
| MVPA (minutes) | 96.6 | 28.1 | 185.5 | 0.8 | 0.3 |
| Sleep (minutes) | 529.3 | 43.0 | 316.5 | 1.0 | -0.2 |
| Mother’s BMI | 26.0 | 2.9 | 25.9 | 5.2 | 1.0 |
| Father’s BMI | 25.0 | 2.8 | 26.0 | 2.4 | 0.6 |
| Screen time (hours) | 3.8 | 2.4 | 10.0 | -0.3 | 0.5 |
| Outdoor time (hours) | 3.0 | 1.5 | 7.7 | 0.7 | 0.7 |
| Level of parental education | 1.3 | 0.6 | 2.0 | 1.6 | 1.6 |
| Mother’s employment status | 1.3 | 0.5 | 1.0 | -1.2 | 0.9 |
| Father’s employment status | 1.5 | 0.5 | 1.0 | -2.0 | -0.1 |
| Commute to school | 1.2 | 0.4 | 1.0 | 1.6 | 1.9 |
| Participation in sports in past year | 1.8 | 0.4 | 1.0 | 0.5 | -1.6 |
| Health-related quality of life | 1.8 | 0.4 | 1.0 | 0.05 | -1.4 |
| Consumption of breakfast | 2.1 | 0.9 | 2.0 | -1.7 | -0.2 |
| Consumption of vegetables | 1.9 | 0.5 | 2.0 | 0.6 | -0.1 |
| Consumption of fast food | 1.2 | 0.4 | 1.0 | -0.01 | 1.4 |
| Consumption of fried food | 1.4 | 0.5 | 1.0 | -1.6 | 0.6 |
| Consumption of fast food watching TV | 1.4 | 0.5 | 1.0 | -1.7 | 0.6 |
| Number of televisions in the house | 1.5 | 0.7 | 2.0 | 0.1 | 1.2 |
| Number of functional cars at home | 1.4 | 0.7 | 2.0 | 0.4 | 1.3 |
| Number of participant’s siblings | 0.6 | 0.5 | 1.0 | -1.8 | -0.5 |
| Number of residents at home | 2.9 | 1.1 | 3.0 | -1.2 | -0.5 |
| High crime rate in the neighbourhood | 1.4 | 0.5 | 1.0 | -1.9 | 0.4 |
| Trust people in the community | 1.6 | 0.5 | 1.0 | -1.9 | -0.4 |
| Urban/rural school location | 1.5 | 0.5 | 1.0 | -2.0 | -0.05 |
| Physical activity policies | 1.6 | 0.5 | 1.0 | -1.7 | -0.5 |
| Healthy eating policies | 1.5 | 0.5 | 1.0 | -2.0 | 0.2 |

SD: Standard Deviation; BMI: Body Mass Index; SED: Total Sedentary Time; MVPA: moderate- to vigorous-intensity physical activity; LAP: Light Physical Activity; TV: Television
